# Responding to the post-pandemic crisis: post-exposure prophylaxis for TB

**DOI:** 10.5588/ijtld.22.0342

**Published:** 2022-09-01

**Authors:** C. Heffernan, R. M. Savić, R. G. Long, M. C. Raviglione, G. Ferrara

**Affiliations:** 1 Division of Pulmonary Medicine, Department of Medicine, Faculty of Medicine and Dentistry, University of Alberta, Edmonton, AB, Canada; 2 Department of Bioengineering and Therapeutic Sciences, University of California San Francisco, San Francisco, California, USA; 3 Alberta Health Services, Edmonton, AB, Canada; 4 Centre for Multidisciplinary Research in Health Science, University of Milan, Milan, Italy

With more than 1.5 million TB deaths every year,^1^ and one quarter of the world’s population infected with *Mycobacterium tuberculosis*,^2^ TB remains a major cause of potentially preventable morbidity and mortality. The WHO End TB Strategy aims to “end TB” by 2035 through integration of the United Nations (UN) Sustainable Development Goals (SDG) 2016–2030.^3^ Treating TB infection (TBI) is considered an essential component of effective TB elimination efforts.^4^,^5^ Specifically, early active case detection and treatment and TBI treatment in high-risk populations are a two-pronged approach critical to achieving the desired decline in TB incidence worldwide.^3^ The 2021 WHO Global TB Report underscored the dramatic consequences of the COVID-19 pandemic: major disruption to TB services worldwide resulted in a significant reduction in diagnostic capacity, notifications and treatment opportunities, and an increase in the number of TB deaths.^1^ Responding to the consequences of the COVID-19 pandemic within the context of national TB programmes will require significant renewed attention, resources and the introduction of bold, new interventions.

The WHO recently published operational guidelines to facilitate integration of TB preventive treatment (TPT) in national control strategies.^6^ This document highlights the importance of preventing active TB and recommends the use, wherever possible, of shorter TBI regimens, including rifapentine (P, RPT) and isoniazid (H, INH) weekly for 3 months (3HP),^7^ or RPT and INH daily for 1 month (1HP).^8^ To note, these guidelines downplay the role of TBI testing, previously considered indispensable for the majority of contacts, and places more emphasis on overall TPT completion.^6^ Although this interpretation removes obstacles to increase its impact in national programmes, the effectiveness of TPT continues to be hampered by several factors.

First, diagnosing TBI is a time-consuming and inaccurate process. The available tests are far from perfect, with false negative results in moderate/high-risk groups, potentially leading to fatal outcomes.^6^,^9^ The tuberculin skin test (TST) requires expertise in performing and reading the test, and its specificity is low, especially in persons vaccinated with bacille Calmette Guérin (BCG).^9^ Interferon-gamma release assays (IGRAs) have a better specificity compared to TST,^9^ but their sensitivity is equally impaired by immunosuppression.^10^ Neither TST nor IGRAs can predict progression to active TB, despite high negative predictive values for reactivation.^9^ They do not discern between active TB and infection, and remain positive after treatment completion.^9^ Exogenous reinfection and consequent progression to active disease account for significant transmission in high-incidence settings.^11^ Current diagnostics neither discriminate reinfection, nor provide ways to use reversion of positive IGRAs in practice, despite growing evidence that this might reflect clearance of recently transmitted bacilli by the cellular immune response.^12^ Moreover, contact tracing and testing are resource and time-intensive, with high rate of loss to follow-up, implying additional system-wide costs for national programmes.^6^ Current TPT guidelines therefore recommend selective testing and treatment of people potentially infected with *M. tuberculosis*, considered at high risk of TB progression, recognising that this assessment relies more on the intrinsic risk of the target population than on the predictive value of available diagnostics.^9^ Studies from India have recently questioned this approach, and have advocated for treatment of all household contacts of new TB cases, given the very high prevalence and high risk of reactivation in these settings.^13^,^14^

Second, once TBI is diagnosed, the rate of treatment completion is impacted by the duration of INH-based regimens, healthy-person acceptance and side effects.^15^ Newer regimens shorten treatment duration,^7^,^8^ but the whole intervention remains ineffective in many settings. Studies on the cascade of care for TBI demonstrate that less than 20% of the target population completes TPT.^16^ This is because testing and treating TBI are resource-intensive for healthcare systems and patients, with significant delays in treatment initiation and follow-up; as a result, the effectiveness of the intervention at population level is drastically impaired.^16^,^17^ Among people living with HIV (PLHIV) and in children <5 years, current guidelines recommend initiating TPT without TBI testing in recognition of the risk of poor outcomes (including disseminated TB) and the related risk-benefit ratio.^6^

Third, the current rationale of TB control relies on models based on observational historical studies predicting a low rate of progression to active disease 3–5 years post-infection. The validity of this assumption was recently questioned because several previously unrecognised factors (e.g., HIV infection) influence the occurrence of active TB and were not accounted for in these predictive models.^18^ These new challenges to long-held assumptions are especially relevant to the design of accurate mathematical models, which need to take into account current epidemiological conditions.^18^

Finally, the current approach based on TBI screening implies delays and loss of opportunities: 4–8 weeks post-infection are needed for the conversion of TBI diagnostics.^19^ At that time, up to 50% of the close contacts of infectious TB cases will be TST-positive,^20^ and will start TPT several weeks after the exposure. This delay introduces more variables, such as the possibility to lose contacts, now known to be infected, to follow-up.^16^ In light of the slow progress further aggravated by post-pandemic recovery challenges, it seems unlikely that TB elimination will be achieved with the current tools. There is, therefore, a clear need to challenge the status quo.

Post-exposure prophylaxis (PEP) is defined as any prompt intervention undertaken to prevent illness immediately following exposure to an infectious agent; PEP is standard of care for pathogens such as *Neisseria meningitidis* and HIV. Moreover, a PEP strategy is under investigation for leprosy, another mycobacterial disease.^21^ Interestingly, the first randomised controlled trials (RCT) with INH as chemoprophylaxis among household contacts of newly diagnosed TB cases showed a TST conversion rate reduction in the treatment arm compared to placebo, suggesting interruption of transmission.^22^ Unfortunately, at the time, such a strategy was not scalable due to the long treatment duration (1 year) and the related hepatotoxicity of INH. Consequently, TBI screening based on TST became the standard of care.^9^ Drawing on the successes of other PEP interventions, a PEP strategy for contacts of active TB cases (PEP-TB) may prove feasible and effective. In close immunocompetent contacts, pre-test probability for TBI is high,^20^ and newer TBI regimens have acceptable safety and tolerability profiles.^7^ This suggests that overtreating potentially non-infected subjects for a short period of time will not be as dangerous as it was with long-term INH. Moreover, active TB could be ruled out relatively inexpensively before PEP-TB is administered.^6^

Recent research supports the PEP-TB rationale. In vitro studies have shown that latent *M. tuberculosis* bacilli are less susceptible to antibiotics,^23^ suggesting that treating for a shorter time immediately after a presumed exposure to *M. tuberculosis* could heal the infection before the latency phase. On this note, RPT daily alone for 6 weeks (6wP) was equally effective as 3HP in animal models and is currently being evaluated in a Phase 3 Clinical trial (NCT03474029), promising to further improve the tolerability profile of TBI treatments.^24^ A cluster randomised trial (CRT)^25^ among household TB contacts would provide the best evidence for PEP-TB (Table). Households where a smear-positive TB case was recently diagnosed can be randomised as clusters between two trial arms, i.e., PEP-TB vs. standard of care (screening of TBI and treatment of only TST/IGRA-positive contacts). The CRT design is preferable to a RCT to avoid contamination^25^ (e.g., members of the same household could be offered different strategies if randomised individually). Those not offered, or declining treatment, will continue to be in the cluster. This design will allow the capture of secondary cases among these subjects, who are excluded in traditional RCTs, but important in perpetuating the transmission of infection (Figure).

**Figure:**
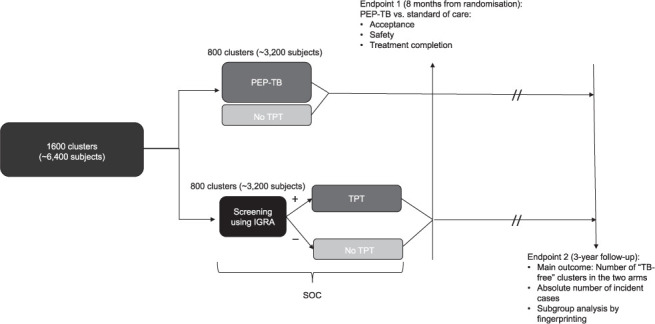
Diagram showing the design of a potential cluster randomised trial for PEP-TB vs. SOC. Definitions: cluster: the entire household of smear-positive TB cases; PEP-TB: post-exposure prophylaxis in TB; SOC: standard of care, treatment of only those individuals who were identified as infected with M. tuberculosis, after a standard screening for infection;^6^,^9^ 3HP: rifapentine (600 mg, 900 mg if weight >50 kg) plus isoniazid (15 mg/kg, max dose 900 mg) once weekly for 12 weeks; no TPT: subjects not treated for any reason (negative IGRA in the SOC; refusal to be treated, allergy to drugs, failure to complete treatment in both arms); TB-free cluster: cluster in which no secondary incident case of TB will occur within the 3 years of follow-up. All clusters of the study will include the subjects under the “no TPT” category. TPT = TB preventive treatment; IGRA = interferon-γ release assay.

**Table:** A potential cluster randomised trial for post-exposure prophylaxis for TB vs. standard of care.

Study design: CRT comparing PEP-TB vs. standard of care (TPT following standard TBI screening) in households (cluster) where a smear-positive TB case was recently diagnosed
Inclusion criteria: Entire households (clusters) of smear-positive TB cases
Exclusion criteria: Suspicion of active disease according to standard guidelines;^5^ presence of resistance to rifampicin (demonstrated using Xpert^®^ MTB/RIF; Cepheid, Sunnyvale, CA, USA)
Interventions: clusters will be randomised 1:1 to: PEP-TB: treatment of the whole cluster with 3HP; treatment start as close as possible to the notification of the index case. An IGRA will be obtained in all participants, but the result will remain blinded to participants and investigators Standard of care: TPT with 3HP, following conventional contact investigation with IGRAs. Treatment start within 3 months from the notification of the index case, only in the subjects deemed infected^5^,^8^
Subjects aged <14 years and other subjects not eligible for 3HP will be treated with other available and suitable regimens in both arms
Patient reported experience measures for acceptability will be obtained at the end of treatment in both arms
The sample size for the trial is estimated at 1,600 clusters (~6,400 household contacts)
All index and secondary cases will be genotyped with whole-genome sequencing
Main outcomes: number of “TB-free” clusters at 3 years after the exposure to the index case in the two arms. Within-clusters transmission will be proven by genotyping of the culture samples obtained from index and incident cases
Secondary outcomes: Total number of incident cases, acceptability, safety and treatment completion in the two arms. Subgroup analysis for genotyping status will be performed in order to prove in-household transmission
Expected results: PEP-TB will be superior to standard of care, by effectively reducing the occurrence of incident cases within the clusters, with similar safety, shorter time to treatment initiation, higher acceptance and completion rate

CRT = cluster randomised trial; PEP-TB = post-exposure prophylaxis in TB; TPT = TB preventive treatment; TBI = TB infection; 3HP = rifapentine (600 mg, 900 mg if weight >50 kg) plus isoniazid (15 mg/kg, max dose 900 mg) once weekly for 12 weeks; IGRA = interferon-γ release assays.

PEP-TB for household TB contacts may prove superior to the standard of care in preventing new cases (resulting in “TB-free” clusters), similarly safe, with higher completion and patient acceptability, while simultaneously eliminating labour-intensive and expensive TBI screening. The primary outcome is the number of “TB-free” clusters at the end of the study in the two arms. Potential secondary outcomes and subgroup analyses are illustrated in the Table and Figure. An open-label trial enrolling 40 household contacts of smear positive TB cases, treated with 3HP in a PEP-TB fashion could test feasibility of the approach before a properly powered CRT. A protocol and a consortium for the CRT could subsequently be developed and submitted for application to the appropriate research ethical boards for surveillance. If the CRT demonstrates its superiority and safety, PEP-TB will represent a powerful new tool to reach TB elimination^3^,^4^ in the post-pandemic era.
